# Evidence of a putative glycosaminoglycan binding site on the glycosylated SARS-CoV-2 spike protein N-terminal domain

**DOI:** 10.1016/j.csbj.2021.05.002

**Published:** 2021-05-04

**Authors:** Zachariah P. Schuurs, Edward Hammond, Stefano Elli, Timothy R. Rudd, Courtney J. Mycroft-West, Marcelo A. Lima, Mark A. Skidmore, Richard Karlsson, Yen-Hsi Chen, Ieva Bagdonaite, Zhang Yang, Yassir A. Ahmed, Derek J. Richard, Jeremy Turnbull, Vito Ferro, Deirdre R. Coombe, Neha S. Gandhi

**Affiliations:** a QUT, Centre for Genomics and Personalised Health, Cancer and Ageing Research Program, School of Chemistry and Physics, Faculty of Science and Engineering, Institute of Health and Biomedical Innovation, 2 George Street, Brisbane, QLD 4000, Australia; b Zucero Therapeutics Ltd, 1 Westlink Court, Brisbane, Queensland, Australia; c Istituto di Ricerche Chimiche e Biochimiche “G.Ronzoni”, via Giuseppe Colombo 81, 20133 Milano, Italy; d National Institute for Biological Standards and Control, Analytical and Biological Sciences Division, Blanche Lane, South Mimms, Potters Bar, Hertfordshire EN6 3QG, UK; e Molecular & Structural Biosciences, School of Life Sciences, Keele University, Newcastle-Under-Lyme, Staffordshire ST5 5BG, UK; f Copenhagen Center for Glycomics, Department of Cellular & Molecular Medicine, University of Copenhagen, Copenhagen N 2200, Denmark; g Department of Biochemistry and Systems Biology, Institute of Systems, Molecular and Integrative Biology, University of Liverpool, Liverpool L69 7ZB, UK; h QUT, Centre for Genomics and Personalised Health, Cancer & Ageing Research Program, Institute of Health and Biomedical Innovation at the Translational Research Institute (TRI), 37 Kent Street, Woolloongabba, Queensland 4102, Australia; i School of Chemistry and Molecular Biosciences, The University of Queensland, Brisbane, QLD 4072, Australia; j Australian Infectious Diseases Research Centre, The University of Queensland, Brisbane, QLD 4072, Australia

**Keywords:** Heparan sulfate, SARS-CoV-2, COVID-19, Spike protein, Heparin, Coronavirus, Cosolvent MD simulations

## Abstract

SARS-CoV-2 has rapidly spread throughout the world’s population since its initial discovery in 2019. The virus infects cells via a glycosylated spike protein located on its surface. The protein primarily binds to the angiotensin-converting enzyme-2 (ACE2) receptor, using glycosaminoglycans (GAGs) as co-receptors. Here, we performed bioinformatics and molecular dynamics simulations of the spike protein to investigate the existence of additional GAG binding sites on the receptor-binding domain (RBD), separate from previously reported heparin-binding sites. A putative GAG binding site in the N-terminal domain (NTD) of the protein was identified, encompassing residues 245–246. We hypothesized that GAGs of a sufficient length might bridge the gap between this site and the PRRARS furin cleavage site, including the mutation S247R. Docking studies using GlycoTorch Vina and subsequent MD simulations of the spike trimer in the presence of dodecasaccharides of the GAGs heparin and heparan sulfate supported this possibility. The heparan sulfate chain bridged the gap, binding the furin cleavage site and S247R. In contrast, the heparin chain bound the furin cleavage site and surrounding glycosylation structures, but not S247R. These findings identify a site in the spike protein that favors heparan sulfate binding that may be particularly pertinent for a better understanding of the recent UK and South African strains. This will also assist in future targeted therapy programs that could include repurposing clinical heparan sulfate mimetics.

## Highlights

• Co-solvent MD simulation identified a putative glycosaminoglycan binding site.
• Novel binding site identified at R246 and the mutated S247R bind glycosaminoglycans.
• Heparan sulphate bridges the site between R246-S247R and PRRAR furin cleavage site.
• Heparan sulphate interacts with H69, pertinent to the UK strain.

## Graphical abstract

**Figure:**
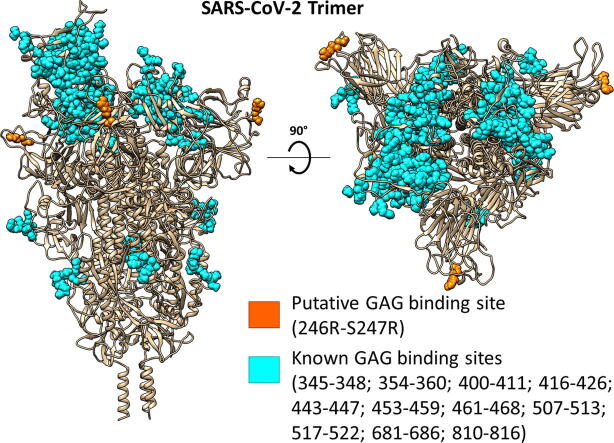

## 1 Introduction

The rapid spread of SARS coronavirus 2 (SARS-CoV-2) since its appearance in late 2019 has elicited a swift response from the scientific community to develop treatments and vaccines against this virus. Effective and efficient drug discovery requires understanding the molecular events of the virus infection pathway and knowledge of the effects of the virus on host immunity. SARS-CoV-2 is an enveloped positive-sense RNA virus, one of several coronaviruses (*Coronaviridae*) that cause respiratory infections in humans. Before the emergence of SARS-CoV-2, there were two highly pathogenic coronaviruses, SARS-CoV and MERS-CoV, which caused severe respiratory disease in humans, and four other human coronaviruses (HCoV-OC43, HCoV-229E, HCoV-NL63, HCoV-HKU1) which induced mild upper respiratory disease. SARS-CoV-2 is closely related to SARS-CoV and MERS-CoV, and, like these two viruses, it can cause very severe disease in some patients. Although the mutation rate of SARS-CoV-2 is considered moderate compared with other RNA viruses, numerous variants have been recorded; some of these have mutations in the spike (S) glycoprotein of the virus outer surface [1], the glycoprotein that is involved in virus infection of cells. The role of the SARS-CoV-2 S glycoprotein in virus infection makes it a key target for the development of antiviral drugs and vaccines. Accordingly, understanding how particular mutations in this glycoprotein may impact virus infection is a key part of the drug discovery process.

**Fig. 1:**
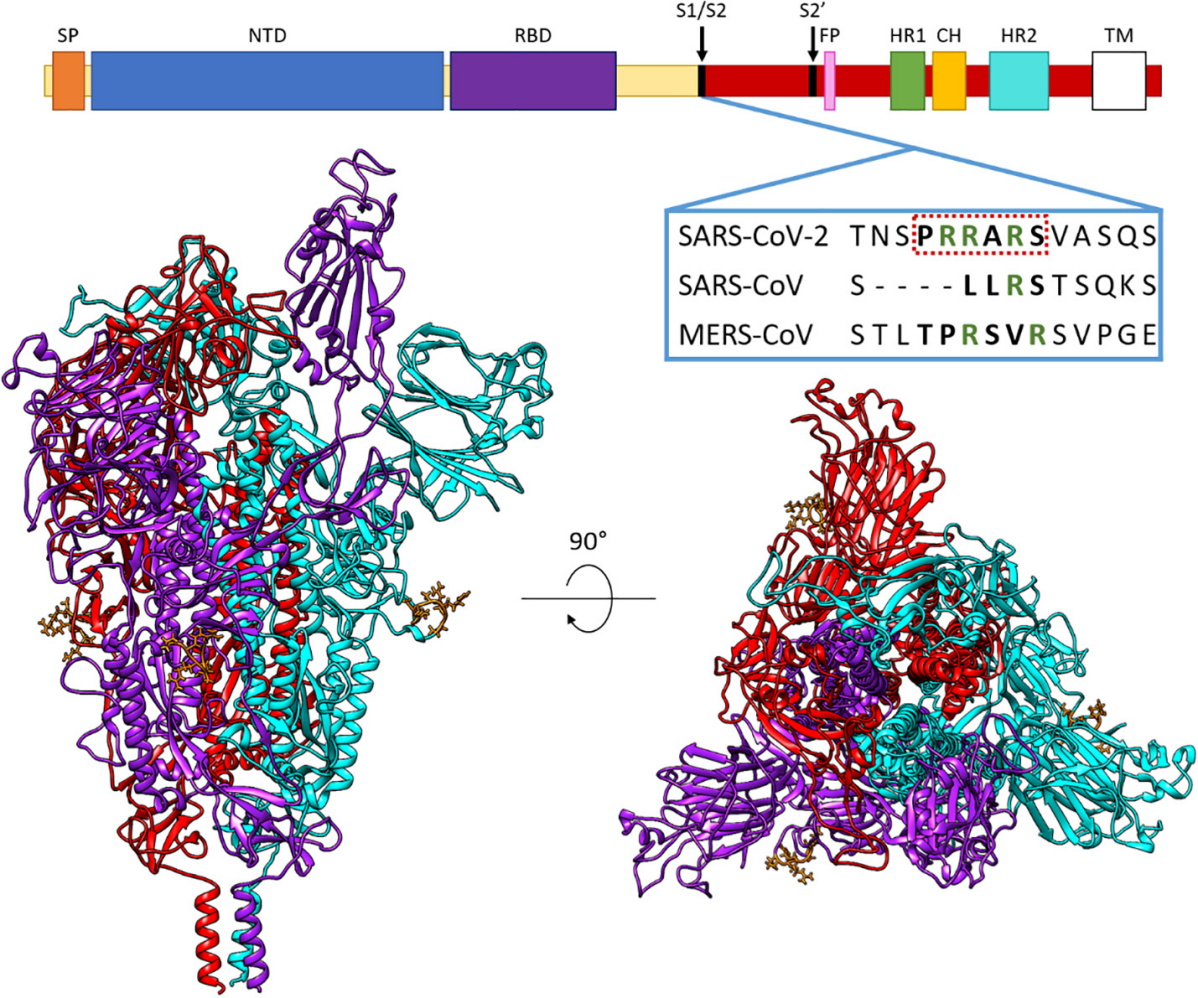
The main domains of the SARS-CoV-2 spike protein, highlighting the unique polybasic furin cleavage site that doubly functions as a GAG-binding motif, absent in the aligned SARS-CoV and MERS-CoV. Annotated domains include SP, signal peptide; NTD, N-terminal domain; RBD, receptor binding domain; S1/S2, cleavage site between S1 and S2 domains; FP, fusion peptide; HR1, heptad repeat 1; CH, central helix; HR2, heptad repeat 2; TM, transmembrane domain. The spike trimer structure was built from PDB: 6VSB and 6LZG. The up conformation of RBD is in purple; the down conformations in cyan and red; and the PRRARS domain is in orange. (For interpretation of the references to colour in this figure legend, the reader is referred to the web version of this article.)

The SARS-CoV-2 S glycoprotein forms homotrimers on the virus surface where it is involved in the multistep receptor-mediated pathway of virus-host cell adhesion and virus-host cell membrane fusion, which culminates in cell infection. The S glycoproteins of other *Coronaviridae* family members also perform this role, but not all coronaviruses that are human pathogens recognize the same cell surface receptor. The cell surface receptor for SARS-CoV-2 is the human angiotensin-converting enzyme 2 (ACE2). Each S glycoprotein monomer consists of two main functional domains – S1 (residues 14–685) and S2 (residues 686–1273) (Fig. 1). It is well established that the S1 subunit mediates virus attachment to epithelial and other cell surfaces by binding to its receptor ACE2, while the S2 subunit mediates the fusion of the viral and human cell membranes [2], [3]. Within S1, a region spanning residues 333–527 constitutes the receptor-binding domain (RBD) [4]. Cryo-EM studies revealed that the S glycoprotein trimer exists in several different conformational states. A significant fraction of the trimers are in a state with one of the three RBDs in an “up” or “open” conformation, whereas other trimers had the RBDs “down” or “closed” [2], [5], [6]. This has the effect of either masking (closed conformation) or exposing (open conformation) the ACE2 recognition interface on the RBD. The factors driving the conformational change are unclear, although a recent study suggested that interactions of the S glycoprotein with the glycosaminoglycans (GAGs), heparan sulfate (HS), or the structurally related heparin (HP), could be a contributing factor [7]. Specifically, it was found that the S glycoprotein could bind HP and ACE2 simultaneously. Moreover, HP oligomers enhance the binding of S glycoprotein to ACE2 [7]. Modelling studies led to the proposal that the site where HP and HS binds on the RBD is partially obscured in the closed conformation but completely exposed in the open state. These findings suggested that HP binding might increase the proportion of trimers in an open conformation, thereby assisting ACE2 binding [7]. Data from the same study indicated that HS side chains of cell surface proteoglycans (HSPGs) are necessary co-factors for infection by SARS-CoV-2, as removing cell surface HS with heparin lyases dramatically reduced S glycoprotein binding to cell surfaces, and SARS-CoV-2 infection [7]. Furthermore, another study found that whilst ACE2 is the primary receptor, the S glycoprotein can interact with cell surfaces in the absence of ACE2, suggesting that the initial interaction is independent of ACE2 [8].

Upon successful attachment to the cell surface, the S glycoprotein is cleaved by serine endoproteases at the S1/S2 site and at the S2′ site (Fig. 1). These events cause the dissociation of the S1 domain and a significant conformational change within S2, which brings the host and viral membranes together leading to fusion of the membrane bilayers and viral entry into the cell. This is a mechanism common to coronaviruses [9]. For SARS-CoV-2, the enzymes involved are believed to be the proprotein convertase, furin, and transmembrane serine protease 2 (TMPRSS2) [10]. Inhibitors of furin cleavage and TMPRSS2 cleavage have been shown to block virus entry and suppress virus production [11], [12].

Heparan sulfate is a ubiquitous component of both the glycocalyx on cell surfaces and the extracellular matrix (ECM) that surrounds and supports cells in tissues. The ECM and a cell’s glycocalyx must be traversed before viruses, and infectious organisms can engage their cell-surface receptors to mediate their entry into cells. In addition to SARS-CoV-2, the related coronaviruses SARS-CoV and HCoV-NL63 also use HS as attachment factors to facilitate binding to their receptor, ACE2, and infection of cells [13], [14], [15]. Glycosaminoglycans are a family of anionic carbohydrates of which HS and HP are members. Both these GAGs are linear polysaccharides of repeating disaccharides consisting of an *N*-acetylated or *N*-sulfated glucosamine alternating with a uronic acid that is either a D-glucuronic acid or an L-iduronic acid [16], [17]. Each monosaccharide in the repeating region of HP and HS may also possess varying degrees of sulfation due to further modifications during biosynthesis. Generally, epimerization of glucuronic acid to iduronic acid is relatively low in HS, whereas for HP the reverse is true. In HP, most disaccharides carry near complete modifications (except of the rare 3-*O*-sulfate) with iduronic acid residues sulfated at carbon-2 (2S), alternating with *N*-sulfated glucosamine that is *O*-sulfated at carbon-6 (6S). These tri-sulfated disaccharides account for ~70–80% of HP. In contrast, approximately ~ 50% of HS chains are composed of unsulfated D-glucuronic acid β(1 → 4) *N*-acetylated D-glucosamine. In HS, there are also highly sulfated regions containing tri-sulfated disaccharides, such as L-iduronic acid (2S) α(1 → 4) D-*N*-sulfoglucosamine (6S), amongst other sulfated variants. The rare structure of 3-*O*-sulfated, *N*-sulfated, and 6-*O*-sulfated glucosamine, can occur in the highly sulfated regions of both HS and HP [17]. The unsulfated and highly sulfated regions of HS are typically flanked by transition regions which consist of partially modified disaccharides separated by completely unmodified *N*-acetylated domains. This domain structure is a dominant feature of HS chains that is largely lacking in heparin chains. It is an important factor of HS-protein interactions due to the flexibility of the non-sulfated regions. Together with variations in their underlying disaccharide structure, HS and HP chains also vary in length and collectively these variations in HS and HP chains give rise to structures that are extremely heterogeneous. The SARS-CoV-2 spike protein displays a higher binding affinity to certain HS or HP sulfation patterns [18]. Chain-length and 6-*O*-sulfation have the greatest effect on affinity [18], [19].

The S protein is heavily glycosylated, with the *N*-glycans attached to it accounting for up to half of the protein’s molecular weight. It is likely that glycosylation shields the amino acid residues and epitopes of the S protein from recognition by cells and antibodies [2]. This probably impairs the host's ability to raise an adaptive immune response targeting the S protein, as generally glycans are poor immunogens. Glycosylation may also increase the infectivity of the virus. To date, most computational simulations of the spike trimer in the presence of ligands/co-receptors have ignored its glycosylation despite readily available information on the glycosylation pattern [19], [20], [21], [22], [23], [24], [25], [26]. Understanding the glycosylation pattern is crucial when developing inhibitors of a protein, as the glycosylation chains can sterically hinder the binding of candidate ligands [24]. Ideally, therapies should target accessible surface regions on the coronavirus that are highly conserved and unlikely to mutate [27]. Hence, in this study we carried out MD simulations that considered the glycosylation.

Most studies looking to develop treatments like antibodies [28], [29], [30], repurposed drugs [31], GAGs [18], [32], [33], or fatty acid-like molecules [34], have been targeting RBD sites within the S glycoprotein. However, it has been determined that mutations distal from the RBD influence the transmissibility of SARS-CoV-2. One mutation that has attracted interest is D614G [35]. This mutation appears to increase viral infectivity by favoring the open RBD conformational state, and it is now a major virus variant globally [36], [37], [38]. Therefore, it is astute to study new mutations that are appearing, such as mutations to basic amino acids. One such mutation – S247R – was documented in the first patient diagnosed with COVID-19 in Australia (GenBank: QHR84449.1). Basic residues have a greater affinity for GAG molecules, as the negatively charged sulfates and carboxylates of the GAG chains can interact with the positive charges of appropriately positioned basic amino acids [39]. The S1/S2 boundary of SARS-CoV-2 glycoprotein contains a unique polybasic furin cleavage site PRRARS (681–686). This site does not appear in SARS-CoV-1 or MERS [2] (Fig. 1) and is one of several suggested GAG-binding motifs in the S glycoprotein from analyses of the amino acid sequence. Another non-RBD HP binding sequence is located at 810–816 (SKPSKRS) [19], but this is not exposed for binding to GAGs on monomers in either the up or down conformations. The sequence YRLFRKS is in the RBD domain and binds short oligosaccharides. To date, most predicted HP binding sites are located in the RBD. These include amino acid sequences 345–348; 354–360; 400–411; 416–426; 443–447; 453–459; 461–468; 507–513; 517–522 and 681–686. These sites have been published by Kim et al. [19], Mycroft-West et al*.* [33] and Paiardi et al. [40].The PRRARS site is exposed, and it appears to contribute to the S glycoprotein-ACE2 interaction in an auxiliary role through electrostatic interactions and its effects on hydration [21]. Importantly, cleavage at this site by furin or related proprotein convertases is essential for activating the S glycoprotein for its role in the fusion of virus and cell membranes, thereby contributing to virus production and syncytium formation [41].

Many studies have focused on the structure and function of SARS-CoV-2-neutralizing antibodies targeting the association of ACE2 and the S glycoprotein RBD domain [5], [30], [42], [43], [44], [45], [46]. The S glycoprotein can rapidly accumulate escape mutations in an experimental system using a replicating VSV-SARS-CoV-2-S virus under selection with a single antibody targeting the RBD. To avoid loss of antibody binding affinity and drug efficacy because of mutations in the RBD, alternatives such as using antibody cocktails as therapies are being investigated [47], [48]. Of particular interest are some antibodies that have been experimentally shown to bind to non-RBD sites of the S glycoprotein. The antibody 4A8 demonstrates potent neutralizing activity. It binds to the NTD residues (R246, Y145-K147, N149, K150, W152) that restrain conformational changes (PDB: 7C2L) [49]. [49]The antibody 2G12 binds to a region that includes the furin cleavage site but does not neutralize the spike protein (PDB: 7L06) [50].

Understanding the dynamic structure of the S protein provides insight into the molecular mechanisms of the protein and its recognition by the host immune system, and it may reveal potential therapeutic intervention points. Recent studies have shown heparin binding to the RBD domain [33], [51] and furin cleavage sites [19]; however, binding sites appropriate for GAG oligosaccharides longer than an octasaccharide remain elusive. Here, using structural bioinformatics methods, docking, and molecular dynamics simulations (MD), we identified HS binding sites on the NTD of the S protein. We then studied, in the presence of glycosylation, the interaction of HS and HP molecules with the S247R mutant protein, a variant of the S protein that first emerged in Melbourne, Australia (GenBank: QHR84449.1) [52]. We propose longer GAG molecules are able to bridge the gap between the PRRARS furin cleavage site and the 245H-246R site with the S247R mutation improving this binding. The emergence of specific mutations in the S protein of UK B.1.1.7 [53] and South African B.1.351 [54] variants has caused these strains to be more virulent. How these mutant residues contribute to virulence is not yet clear. It is possible that GAG binding to the NTD site we identified may be influenced by some of these mutations and so contribute to the virulence of these new strains. Targeting the new binding site with antibodies or structurally tailored GAG dodecasaccharides (or longer GAGs) capable of bridging the gap between the furin site and residues 245H-246R may prevent proteolytic cleavage at the S1/S2 domain boundary of the S protein, thereby preventing infection of host cells.

## 2 Material and methods

### 2.1 Trimer model building

A complete trimer model was built to investigate GAG oligosaccharide binding sites. To do this, the cryo-EM structure of the trimeric S protein in its prefusion conformation (PDB: 6VSB [6]) was used as the base model. A homology model was built using the SWISS-MODEL webserver [55] to account for missing loops in the PRRARS furin cleavage site and RBD domains in the down conformation. The RBD domain from the RBD-ACE2 crystal structure (PDB: 6LZG [56]) was isolated and used to replace the RBD subunit in the up conformation from the homology model. The combined model was merged using Modeller in UCSF Chimera v. 1.13.1 [57]. While there are now multiple trimer structures available, this was the only trimer model structure available at the time of this work [57].

To run simulations as close to biological conditions as possible, glycosylations and disulfide bonds were added to the model using the CHARMM-GUI web-server Glycan Reader & Modeler input generator [58], [59], [60]. N- and C-termini were treated as being neutral, and disulfide bonds were added between cysteine residues 131–166, 291–301, 336–361, 379–432, 391–525, 480–488, 538–590, 617–649, 662–671, 738–760, 743–749, 840–851, 1032–1043 and 1082–1126. Glycosylations were added according to Table S1, adapted from the site-specific model by Grant et al. [25].

### 2.2 Modelling of spike trimer-heparin tetrasaccharides in cosolvent MD simulations

Cosolvent molecular dynamics (MD) simulations were carried out on the trimeric model described above to search for new oligosaccharide binding locations. During a cosolvent simulation, the protein local environment and bulk are sampled to characterize potential binding sites on the protein surface. In our experiment, ten HP tetrasaccharides with the sequence [IdoA2S-α-(1 → 4)-GlcNS6S-α-(1 → 4)]_2_ were used as the cosolvent. These were randomly placed around the trimer model. The CHARMM-GUI input generator [59], [60] was used to place the protein at the center of a triclinic simulation box, with 15 Å edge space. The box was solvated with TIP3P water [61] and 0.15 M NaCl placed with the Monte-Carlo method to neutralize the system charge. Long-range electrostatics were treated using the particle-mesh Ewald (PME) method. Periodic boundary conditions (PBC) were applied throughout the simulations. A non-bonded cut-off of 12 Å was used and the non-bonded neighbor list was updated at every time step. The SHAKE algorithm [62] was used to constrain all bonds involving hydrogen atoms. Minimization, equilibration and production simulations were performed with GROMACS v. 2020.1 [63], [64] using the CHARMM36 forcefield [65] on V100 GPU of the NCI Gadi supercomputer. The system was first minimized for 5,000 steps followed by equilibration for 10 ns with 2 fs steps. The final production simulation was run for 300 ns, saving a snapshot each 10 ps. Temperature coupling was at 303.15 K and used the Nose-Hoover extended ensemble. Pressure coupling used the Parrinello-Rahman extended ensemble at 1 bar. The final snapshot was analyzed using VMD v. 1.9.3 [66] to determine the locations on the trimer where HP molecules bound. To confirm the results, a second simulation on the same system was conducted for 100 ns starting with different velocities, and a third saturated system with 20 HP tetrasaccharides was built and run for 100 ns with the same settings as described above.

### 2.3 HS and HP ligand docking and MD simulation to S glycoprotein monomer

It is understood that HP favors binding to basic residues. This rationalized a search for mutations involving basic residues that have appeared within global SARS-CoV-2 spike amino acid sequences. The sequences published on the COVID-19 Viral Genome Analysis Pipeline were searched for the site mutations. This database has been assembled using data from the Global Initiative on Sharing Avian Influenza Data (GISAID) database [67].

The initial cosolvent simulations formed the rationale behind the docking and subsequent experiments to test the binding of longer HS to the PRRARS site and S247R. To achieve this, the monomer in the up conformation was isolated from the model. We used USCF Chimera v. 1.13.1 [57] to mutate Ser-247 to Arg-247 and built two systems with the HP and HS dodecasaccharides. GlycoTorch Vina [68] was used to perform docking of oligosaccharides. The dodecasaccharide HP ([IdoA2S-α-(1 → 4)-GlcNS6S-α-(1 → 4)]_6_) and HS ([IdoA2S-α-(1 → 4)-GlcNS6S-α-(1 → 4)]_2_-[IdoA-α-(1 → 4)-GlcNAc-α-(1 → 4)]_2_-[IdoA2S-α-(1 → 4)-GlcNS6S-α-(1 → 4)]_2_ were built using the Glycam GAG builder [69], with the IdoA2S residues considered in the ^1^*C*_4_ conformation. The online GlycoTorch tool was used to convert the PDB input files to PDBQT. This tool accounts for ϕ and ψ glycosidic torsions, and contains parameters to model ^2^S_0_ and ^1^C_4_ for iduronic/glucuronic acids [68]. All sulfate and hydroxyl groups, and glycosidic torsion angles were treated as flexible. The box size was 60, 53, 80 centered between the furin cleavage domain and S247R. For docking we used an energy range of 12, an exhaustiveness of 12, chi_cutoff = 1, chi_coeff = 2 and set the number of modes to 100.

The two systems of the S247R S protein monomer with HP and HS dodecasaccharides were prepared for MD simulations using the CHARMM glycan input generator according to the same settings outlined in 2.2. The MD simulations were performed with GROMACS for 100 ns using the methodology as described in section 2.2 above. Due to the computational resources available, only a single MD run was conducted.

#### 2.4 ClusPro Docking

To confirm the MD simulation results, ClusPro [70] was used to dock heparin to an unglycosylated monomer subunit of the S protein in the “up” conformation and with the S247R mutation. This was to confirm the binding sites observed in the MD simulation described in section 2.3. The resulting models were superimposed, visualized and atom contacts determined using ChimeraX version 1.1 [71] (Fig. S2).

### 2.5 Statistical analysis of heparin-binding motifs

The putative GAG binding sequence within the SARS-CoV-2 NTD were analyzed by methods described in Rudd et al. [72]. A brief description of the method follows. In summary, 776 heparin binding proteins, were fragmented into sequences of a minimium of 3 residues with at least one basic amino acid in the following combinations; BXA, BXS, BXP, BXAS, BXAP and BXPS (where, B = basic, X  = hydrophobic, A = acidic, P = polar and S = special). A comparison of these sequences was made with a metric using the Levenshtein distance. This is a measure of similarity between character strings based on the minimum number of insertions, deletions or substitutions that a string needs to undergo to alter one amino acid sequence to match the other. Sequences with a similarity score of greater than 0.7 (70% similarity) were considered highly conserved. Using this same Levenshtein similarity cut-off of 0.7, sets of highly conserved basic amino acid containing sequences extracted from heparin binding proteins were used to support the validity of the proposed binding site in the SARS-CoV-2 S247R mutant S protein NTD.

### 2.6 Trajectory, MM/PBSA analysis and electrostatic potential surface

Tools built into GROMACS were used to process the trajectories, including re-centering, fitting, periodicity treatments and concatenation before analyzing them with an array of tools. MDAnalysis [73], VMD 1.9.3 [66] and USCF Chimera [57] were used to visualize and analyze the output trajectories. The final 50 ns of the HS and HP trajectories was used to calculate per-residue energy decompositions with the g_mmpbsa [74] module compatible with GROMACS distribution. A frame was extracted every 250 ps from each system and a total of 200 frames were used for the MM/PBSA calculations. The system enthalpy is calculated using the molecular mechanics method (MM). The polar part of the solvent effect is obtained by solving the finite difference Poisson-Boltzmann (PB) equation, and the non-polar part is fitted by estimating the surface area that is solvent-accessible (SA). This is demonstrated in Equation (1):

(1) $${\Delta G}_{\mathrm{bind}}={\Delta E}_{\mathrm{MM}}+{\Delta G}_{\mathrm{sol}}-T\Delta S$$

The binding free energy is ${\Delta G}_{\mathrm{bind}}$, and the intramolecular energy under vacuum is represented by ${\Delta E}_{\mathrm{MM}}$. The solvation free energy difference is ${\Delta G}_{\mathrm{sol}}$, which is the sum of polar and non-polar solvation free energies. T is the absolute temperature and ΔS is the change in entropy of the system. We did not carry an out entropy calculations owing to the size of the systems. CHARMM radii were used and an ionic strength of 0.15 M was set for the salt. The outer dielectric constant was set to 80, and solute dielectric constant set to 2. Further details of the MM/PBSA methods have been published by Genheden and Ryde [75].

The electrostatic potential surface was visualized by preparing files with PDB2PQR webserver [76]. The CHARMM forcefield, with an internal dielectric constant of 2 and a constant of 80 for water was used to map the potential on the DelPhi web server [77], [78], using an internal dielectric constant of 2. ChimeraX version 1.1 [79] was used to visualize the electric potential surface.

### 2.7 Differential scanning fluorimetry

Differential scanning fluorimetry (DSF) was conducted on 1 μg S1 in PBS (pH 7.6) with 1.25 X SYPROTM Orange (Invitrogen) in the presence of 100 μg of either unfractionated porcine mucosal heparin (UFH; Celsus) or size defined heparin oligosaccharides (dp 6, 12 or 16), with H_2_O used as a control. Reaction mixtures were made up to a volume of 40 μL, in 96-well qPCR plates (AB Biosystems) before being subjected to DSF using an AB Biosystems, StepOnePlus, qPCR machine with the TAMRA filter employed. Melt curve experiments were performed by increasing the temperature from 25° C to 90° C by 0.5° C increments every 30 s. Following, smoothing (Savitxky-Golay, 2nd-order polynomial, 9 neighbors) the first differential of the melt curves was calculated (Prism 8; GraphPad) and the peaks used to determine the melt temperature (T_m_) of S1 alone or in the presence of UFH or size defined oligosaccharides (MatLab software; R20018a, MathWorks).

## 3 Results and discussion

**Fig. 2:**
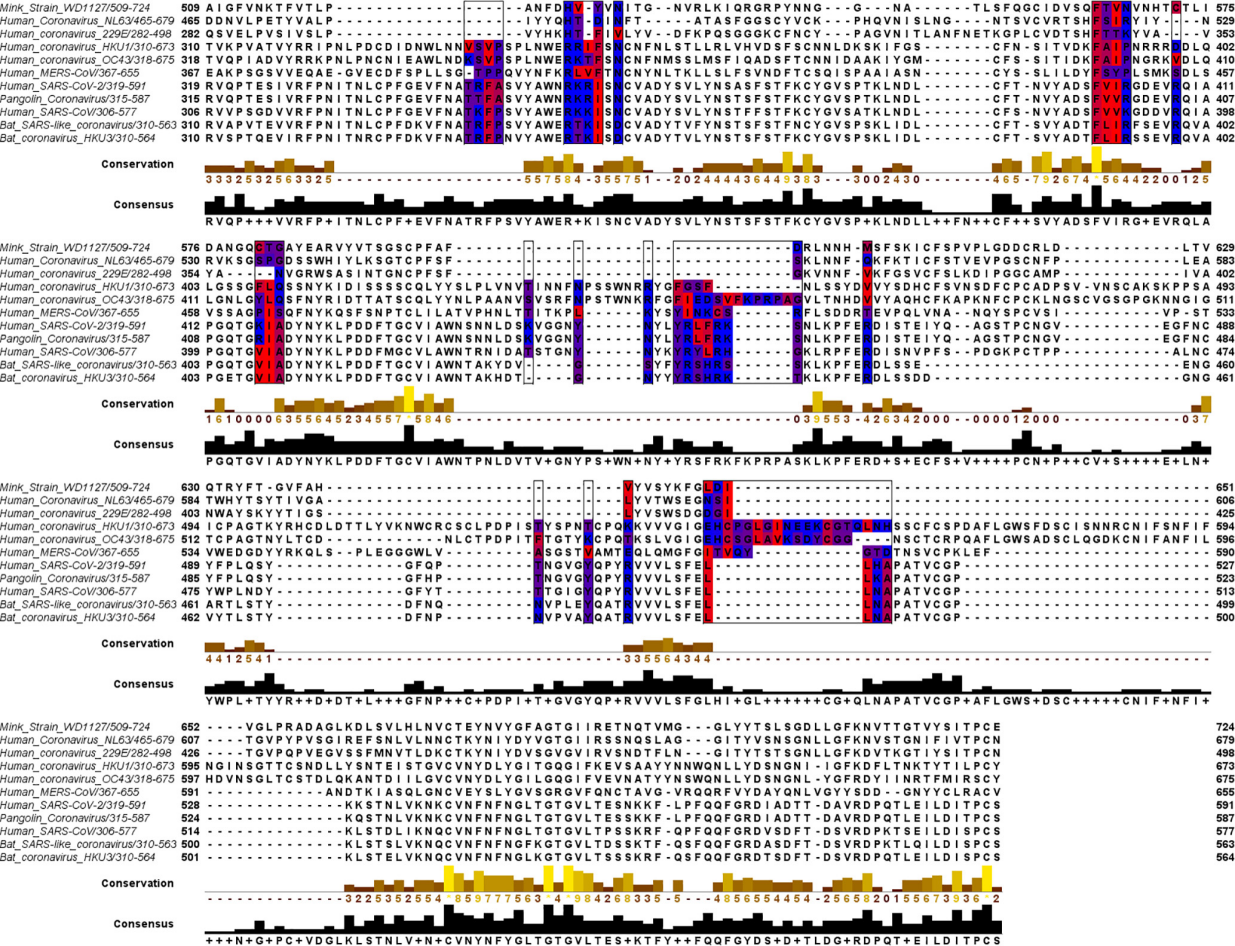
Multiple sequence alignment using Clustal Omega of the RBD of related virus strains. Heparin binding sites identified in previous papers [19], [33] are highlighted and colored according to hydrophobicity in Jalview [101]. Bat, mink, and pangolin strains were compared to the human coronaviruses, as these species are theorized to be the zoonotic hosts of the viruses [80], [81].

**Fig. 3:**
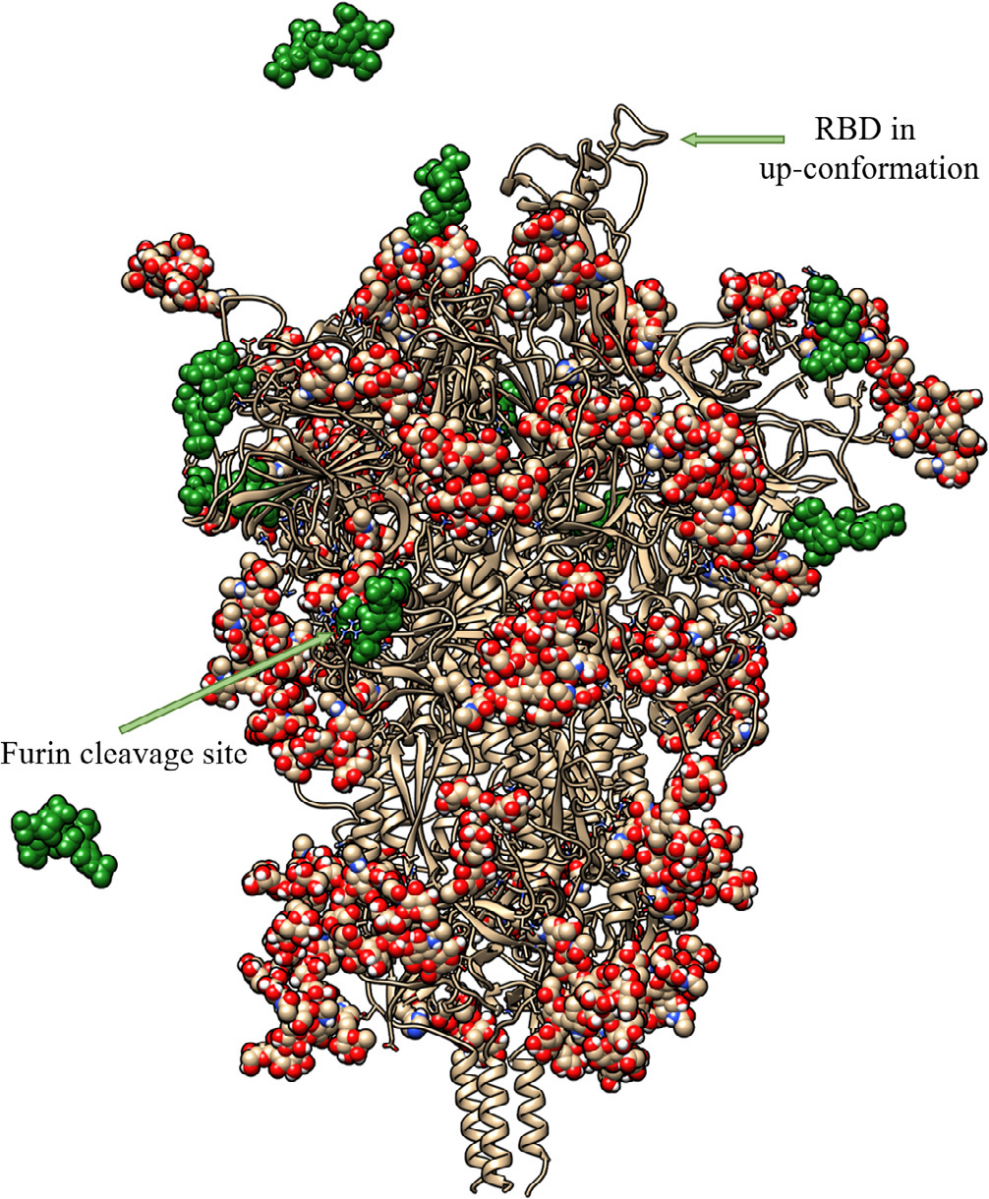
A representative snapshot showing the binding of heparin tetrasaccharides to the glycosylated SARS-CoV-2 S protein, obtained from the unbiased MD simulations. The tan ribbons are the representation of the protein; the glycosylations are the spheres coloured.

It has been reported that the interaction of HS with SARS-CoV-2 is a requirement for the virus to infect cells [7]. The key binding sites on the S protein reported in this earlier study were all located in the RBD domain near the ACE2 binding site. Studies investigating where HP binds to the spike protein have predicted multiple binding sites on the RBD [19], [33], [40]. The RBD protein sequence of several coronavirus strains theorized to be zoonotically related to SARS-CoV-2 [80], [81] were aligned to allow the amino acid sequences of these predicted HP binding sites to be compared. This alignment (Fig. 2) revealed relative conservation of the HP binding sites between viral strains examined. To further understand HS interactions with the SARS-CoV-2 S trimer, we first conducted an unbiased molecular dynamics (MD) simulation of a glycosylated SARS-CoV-2 S trimer with ten HP tetrasaccharides randomly placed around the protein. Such cosolvent MD simulations have literature precedence to detect novel binding sites [82]. Other published computational studies (using CHARMM [83], or Glycam [25] force fields) looked at glycosylation patterns that included N- and O-linked glycoforms [83] and glycoforms in the Golgi prior to enzymatic modification [25], but for the purposes of this study we considered the model used in MD simulations by Grant et al*.* [25]. In our study, seven of the ten HP tetrasaccharides bound to the trimer during the initial MD simulation (Fig. 3). Regions where the HP tetrasaccharides bound to the protein contained the positively charged residues arginine (R246, 249, 577, 634, 682, 683, 685), lysine (K147, 150, 444), and a protonated histidine (H245), as well as the polar uncharged serine (S71), and the small non-polar residues glycine (G72) and alanine (A684). Two further short cosolvent simulations (100 ns) produced the same results, providing good evidence for the binding of HP tetrasaccharides to R246 and the PRRARS domain (Fig. S1). These regions in the S1 NTD give access to the tetrasaccharides to the protein surface between the glycosylation sites.

A common method to provide support to data from MD simulations of GAGs binding to a protein is to dock a generic HP tetramer using the online server ClusPro [70]. We docked heparin to the unglycosylated S247R monomer in the “up” conformation. HP molecules bound to R246-S247R and the furin cleavage site (Fig. S2). This aligns with the results we observed in the MD simulations of the trimer and monomer.

Interestingly, a few of the amino acid residues found to interact with HS in the MD simulations are insertions in the regions 72–82, 144–147 and 244–246, which are shared by SARS-CoV-2 and the bat coronavirus RatG13, but not SARS-CoV. Moreover, these regions are known to play a role in host receptor binding [84], [85], [86]. Hence, we predict they play a significant role in controlling the binding of SARS-CoV-2 to cells, including the process affecting the conformation of the RBD binding site.

**Fig. 4:**
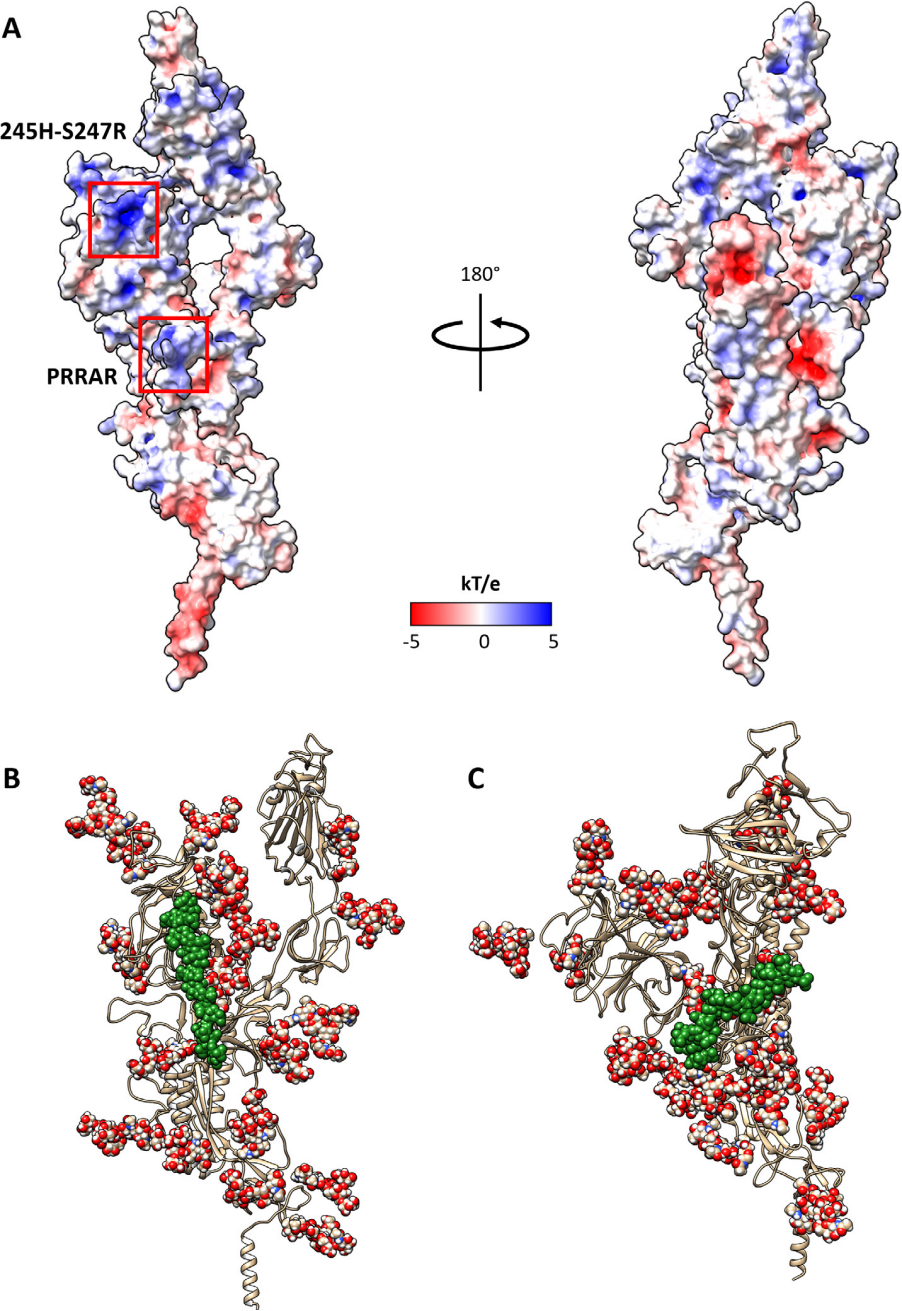
(A) Electrostatic potential surface representation of the spike monomer, calculated using the DelPhi web-server [77], [78]. The left hand side presents the face containing the PRRARS furin cleavage site and 245H-S247R site, while the right hand side is the opposite face. Representative snapshots obtained from MD frames at 100 ns of the (B) HS and the (C) HP dodecasaccharides bound to the S protein in the up conformation. The spheres represent glycosylations and the GAG oligosaccharides as green spheres. (For interpretation of the references to colour in this figure legend, the reader is referred to the web version of this article.)

With mutations to the spike appearing as the pandemic spreads across the globe, we felt it was pertinent to investigate some of these changes because newly arising basic mutations may promote GAG binding. Of particular interest was the S247R mutation, which is physically located close to the PRRARS furin cleavage site and close to some of the deletions in the UK mutant strain (B.1.1.7) (Table S5). The S247R mutation is adjacent to two charged amino acids (H245-R246) that have been targeted by neutralizing antibodies [49]. We hypothesized that the S247R mutation would augment the basic nature of this region containing H245-R246 and favor bridging of GAG molecules between this region and the furin cleavage site. The GAG docking program GlycoTorch Vina [68] was used to test this hypothesis, with HS and HP of different lengths being examined. This determined that GAGs 12 saccharides long, i.e. dodecasaccharides, would bridge the gap in a biased docking (Fig. 4B). An analysis of the electrostatic surface potential (Fig. 4A) revealed that the region HS binds is positively charged. As predicted, the positive region near the H245-R246 is made more basic by the S247R mutation, and this facilitated HS binding.

Using GROMACS [63], [64], we ran MD simulations with the HS and HP dodecasaccharides to conduct an in depth analysis of the binding to the mutated site, S247R, and the PRRARS furin cleavage site. Fig. 4B (HS) and C (HP) show a clear differentiation between the binding modes of the two GAG molecules. Only HS bridges the gap between the furin site and S247R, while HP sits in the pocket between the PRRARS loop and the loop containing R634. The less sulfated HS spans several domains, while HP has limited contact with the S protein as indicated by the distance between residue centers of mass (Fig. S3) according to calculations using MDAnalysis [73], [87]. This analysis also highlighted the residues that appeared to play key roles in binding the oligosaccharides. The distances between the centers of mass are indicative of electrostatic interactions, such as hydrogen bonding, between the atoms involved. The data in Fig. S3 supports previously published data [18] that variation in the sulfation levels of molecules has a significant impact on binding to the S protein. Pulmonary HS is quite heterogeneous [88], with functional studies suggesting it regulates signaling proteins involved in lung development, homeostasis and injury [89]. Given our data, it would be interesting to model the interactions of specific HS structures known to be present in the lung with the S protein to examine whether these structures bind in the way suggested in our model, and so link the furin cleavage site with the region around S247R or H245-R246.

**Fig. 5:**
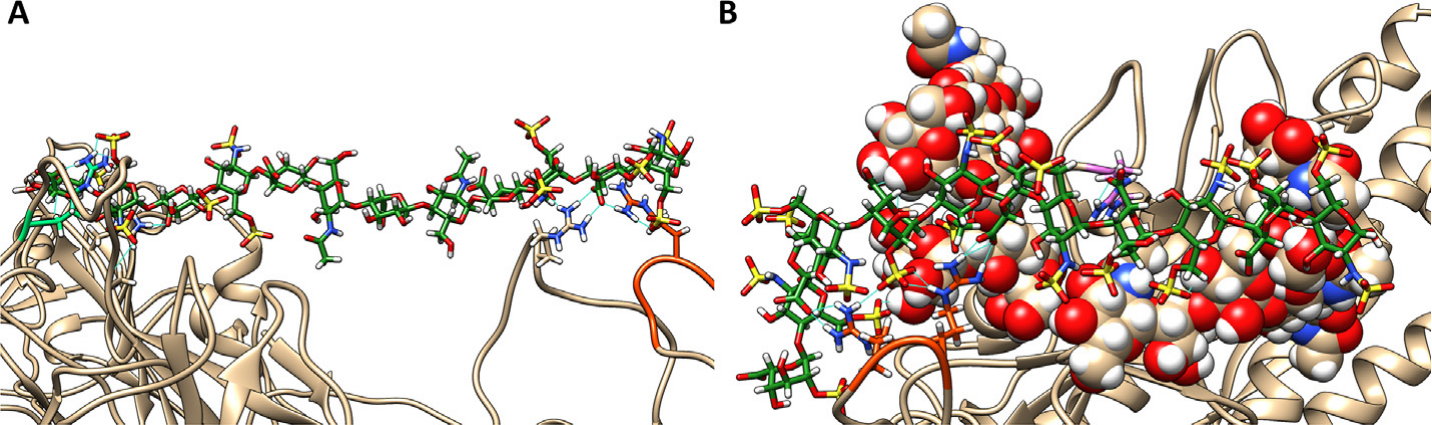
Frames from 100 ns of MD simulations. Hydrogen bonds (solid cyan lines) between residues of the S protein or glycan shield (spheres) and the GAG molecules. The PRRARS site is in orange, and the 247H-S247R is in green. (A) shows the surface binding of the HS molecule to the S247R mutation and residue R683 of the PRRARS domain. Later frames show further binding of PRRARS domain residues. Other residues binding the HS molecule include H69, S71, A262 and R634. (B) shows the hydrogen bonds between the HP molecule and the S protein or glycan shield. Here the residues R683 and R685 of the PRRARS domain form hydrogen bonds. Other residues include R634 and saccharides from the glycan shield. Full information on the hydrogen bonds including residue, atom and distance information are in Table S2 (HS) and S3 (HP). (For interpretation of the references to colour in this figure legend, the reader is referred to the web version of this article.)

**Fig. 6:**
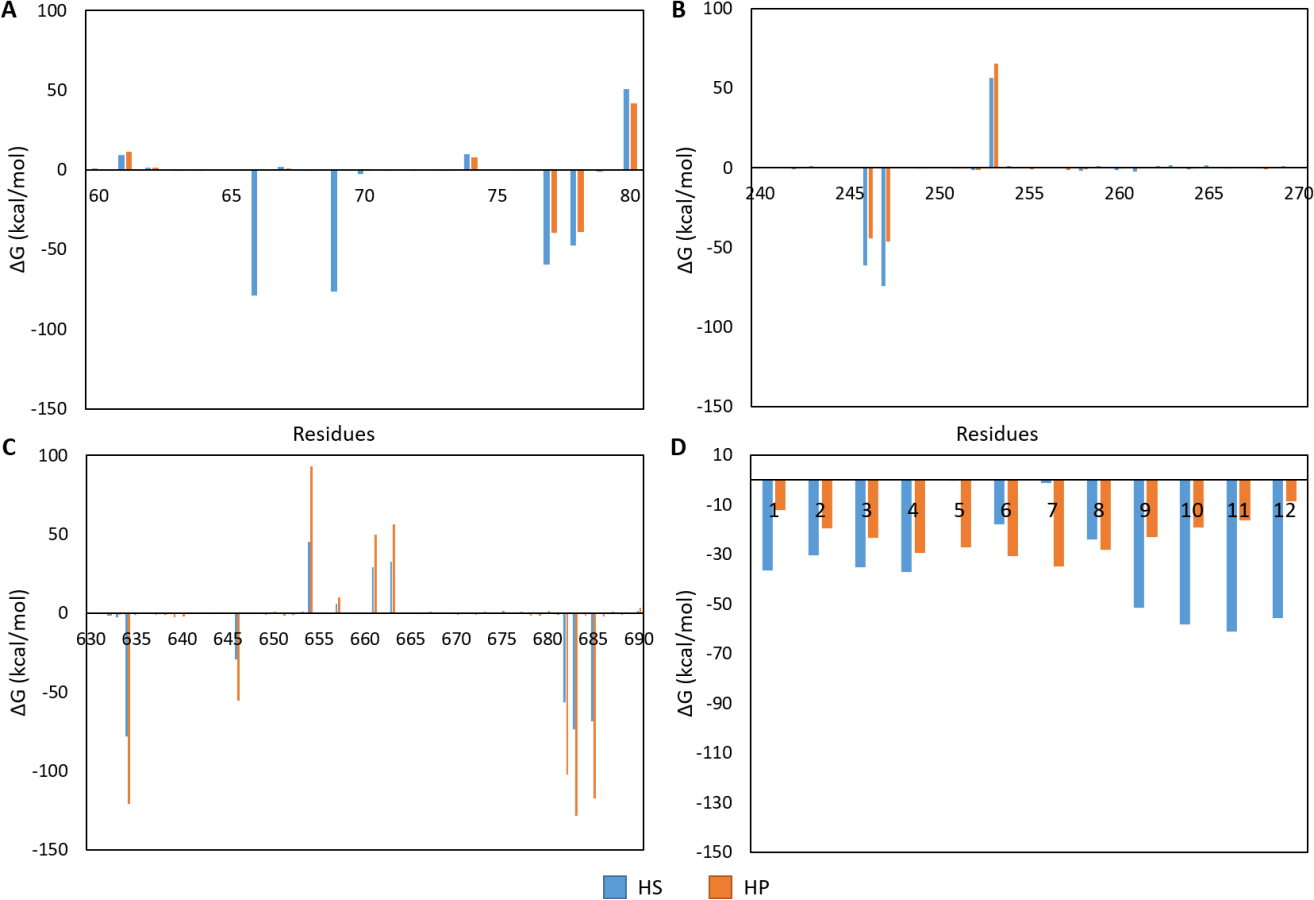
MM/PBSA-binding free energy per residue decomposition [ΔG kcal/mol] for the protein–ligand interactions. The x-axis of each is numbered according to the residue in the protein or GAG. The residues displayed were selected as they were the ones with the largest affinity for the HP/HS, and were therefore of interest. A) the decomposition of the S glycoprotein residues 60–80B) decomposition of the S glycoprotein residues 240–270; C) decomposition of the S glycoprotein residues 630–690; D) decomposition of the HP and HS ligands.

Analysis of the hydrogen bonds formed between the S protein and the two ligands (HS and HP) revealed that glycans on the S protein are involved in binding the HP molecule (Fig. 5). Both HS and HP bind to the PRRARS furin site and interact with R634 (Tables S2 and S3). They otherwise differently bind to the S protein: HS bridges the gap between the PRRARS domain and S247R and does not have any strong interactions with the glycan shield, whereas the N-glycans near the furin cleavage site exert a shielding effect on the short HP dodecasaccharide examined here. Longer HP chains ignore this effect, forming a bridge between RBD heparin binding residues (T345, R346, N354, R355, N360) and the furin cleavage site [40]. This supports the use of UFH and longer heparin chains over LMWH for preventing the binding of SAR-CoV-2 to the cell surface. The higher sulfation density of the HP molecule shifts the preferred binding of this molecule to the PRRARS furin cleavage site and surrounding N-glycans. Compare this to the sulfates of the HS molecule, which were primarily located at either end of the chain, giving rise to a binding mode involving both the furin site and binding to the distal 245H-S247R site. Post-MM/PBSA analysis of the complexes support this (Fig. 6A). In Fig. 6A, HS shows a strong affinity for residues H66 and H69; interestingly H69 is deleted in the recent UK strain (B.1.1.7). Those two histidines are unique in the decomposition as only HS had an effect on them. The residues H66, H69, K77, R78, R246, R247, R634, R646, R682, R683 and R685 were calculated to demonstrate favorable free energy contributions to HS binding, whilst the eight arginines in this list and K77 were found to have favorable free energy for binding to HP (Fig. 6). We believe these differences are a result of a higher flexibility in the non-sulfated region of the HS molecule, compared to the more fully sulfated HP. It is this flexibility that allows the bridging of the two regions (furin cleavage site and the 245H-S247R region), in a manner similar to what was observed in the studies by Perkins [90] and Rashid [91].

A graph showing the decomposition of glycosylations interacting with the ligands is presented in Fig. S4. The figure indicates that the glycosylations on the S protein interact with the HP molecule more than the HS. Specifically, *D*-mannose and *N*-acetylgalactosamine (Table S3) glycan subunits attached to N61 and N616. In our simulation, the glycosylations attached to these residues formed hydrogen bonds with the HP dodecasaccharide. Previous experimental molecular dynamics simulations have evaluated the effects of N-linked glycosylation on serpins and the consequences of heparin binding to both proteases function and dynamics [92], [93], [94]. Similarly, our work alludes to a role that these glycosylations might play in contributing to GAG specificity. This alludes to a role that these glycosylations might play in contributing to GAG specificity.

The differences in the binding of HS and HP highlighted in this present study is in accordance with the results of other studies that indicate more favorable binding to certain lengths and sulfation levels of GAGs [18]. Post-MM/PBSA analysis of the HP- and the HS-S glycoprotein complexes revealed the relative free binding energy and standard deviation of that average to be −461.91 ± 3.97 kcal/mol for the HP complex and −788.39 ± 3.06 kcal/mol for the HS complex.

A key aspect in our approach to identifying novel binding sites on the S glycoprotein is the consideration of the site-specific glycan shield in the MD simulations. This revealed significant interactions between the docked GAG molecules and the glycan side chains, which would not be apparent if these studies had been performed in the absence of S protein glycosylation. In light of this, we were able to identify a gap in the glycan shield at 245H-S247R that may imply an increased viral infectivity of the S247R mutant. At the same time, this new virus strain is likely to bind HS more strongly than the wild type strain. Therefore, its sequestration to the cell surface by proteoglycans like syndecans could be vulnerable to inhibition of by free HS chains or HS fragments. A search of the COVID-19 Viral Genome Analysis Pipeline database revealed other sites of mutation to basic residues. Table S4 shows the number of times such mutations have been recorded. It may be pertinent to investigate these mutations as they could promote the ability of SARS-CoV-2 to bind to cell surfaces, thus increasing virulence. These basic mutations could also act as alternative targets for the development of HP-based therapies. The UK strain B.1.1.7 that is spreading rapidly throughout the UK has a deletion in the NTD at residues 69–70. This is an escape mutation that is believed to have arisen as a result of a treatment regime – in this case, convalescent plasma [95], [96]. Our data identified H69 and S71 to be HS binding residues (Table S1). We have included a commentary of the amino acid residue mutations and their likely roles in HS/HP binding that have occurred in the recent British (B.1.17) and South African (B.1.351) mutant strains of SARS-CoV-2 (Table S5). HS octasaccharides optimally bind to the RBD [97], but it is not yet known where longer HS oligosaccharides bind. As a result of our analyses, we believe they bind to the NTD, whereas the RBD has a higher affinity for shorter oligosaccharides closer to dp8.

The thermal stability of full-length trimeric S1 was investigated using differential scanning fluorimetry (DSF), a technique which utilizes a conventional real time PCR machine to determine the midpoint of unfolding (or melt temperature, T_m_) of a given protein, through the use of a hydrophobic fluorescent probe [98]. When heated, protein unfolding exposes buried hydrophobic regions where the fluorescent dye can bind. This results in an increase in fluorescence that can be used to produce a melt curve. The T_m_ can then be obtained from the peak of the first differential. In this way, protein unfolding can be monitored. The T_m_ of full-length trimeric S1 using this method was determined to be 44° C, approximately 2° C lower than previously reported for the glycosylated RBD of S1 [99].

**Fig. 7:**
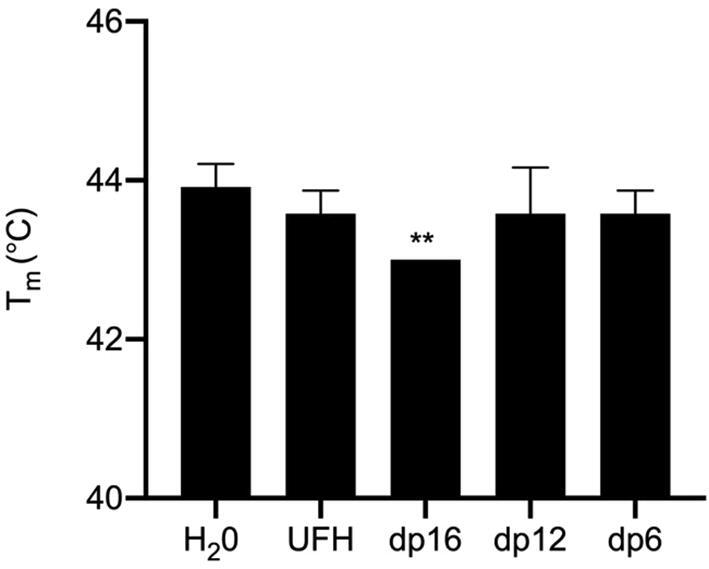
T_m_ of S1 with size defined oligosaccharides or heparin; H_2_O was used as a control for no addition. ** Significant difference between the T_m_ value of S1 H_2_O control (44 °C ± 0.3; n = 3) compared to RBD plus dp16 (43 °C ± 0; n = 3), t(4) = 5.5, p ≤ 0.01 0. ΔT_m_ = 1 °C. Where no error bars are shown SD = 0.

Changes in a protein’s T_m_ value in the presence of a ligand is also indicative of binding affinity, therefore, the ΔT_m_ of S1 with size defined heparin oligosaccharides was determined to investigate the size dependency of binding. Oligosaccharides consisting of 16 saccharide units when mixed with S1 caused a statistically significant 1° C reduction in the T_m_ of S1. Shorter oligosaccharides consisting of 12 or 6 units did not exhibit a statistically significant change in the T_m_ of S1 (Fig. 7), suggesting that heparin chains longer than dodecamers may be required for optimal binding to S1. It is noteworthy that UFH did not show a statistically significant difference in T_m_, thus potentially indicating that there is a limit to the optimal length of HP that S1 can bind or that the sulfation patterns present in the UFH were not optimal for binding.

A comparison of full-length S protein amino acid sequences was made with an extensive library of amino acid sequences from known HP binding proteins. The degree of similarity observed suggests regions on the S protein that are likely to bind to GAGs. The putative binding site at 245H-S247R did not appear with a Levenshtein cut-off of 0.7, but did at 0.65 (Table 1). This is because the sequence 241–246 (LLALHR) extended by the S247R mutation (LLALHRR) is quite rare and not conserved in the library of heparin-binding proteins used for the analysis. The analysis also identified that the 69–71 region is likely to be involved in HP binding.

**Table 1:** Sequence of SARS-CoV-2 S glycoprotein residues with a Levenshtein Distance above 0.7. The normalized count for similar sequences as found in the library of 776 heparin binding proteins. The higher the normalized count, the more often a sequence was identified among the protein set. Basic amino acids are identified; arginine (blue), lysine (red) and histidine (green). AA No; amino acid number. AA; amino acid identity. HBP; relative frequency of sequence among heparin binding proteins. The region highlighted in blue shows residues 241–247 count with a cut-off of 0.65.

## 4 Conclusions

Overall, our work shows the importance of taking into account the glycan shield when conducting MD simulations of proteins, as it can act to prevent binding to certain regions of the protein and directly interact with some docked ligands. Such interactions may be missed when conducting studies without glycosylation being included. By considering the glycan shield, the prediction of important sites involved in molecular interactions and possibly immune recognition is improved. Indeed, our study identified a putative GAG binding site at residues 241–246 of the SARS-CoV-2 S glycoprotein. This is particularly pertinent to the South African strain (B.1.351) mutations of this virus, with its deletion at L242-L244. Finally, this work has confirmed the preferential affinity for particular GAG sulfation patterns and lengths based on bridging between different oligosaccharide binding sites that may act cooperatively as anchoring points to prevent or support conformational changes in the protein. The repurposing of drugs is becoming a common approach to developing novel therapies. This work, among others [7], suggests the potential use against SARS-CoV-2 of HS mimetics such as Sanofi’s SR123781, which reached phase II clinical trials (NCT00123565, NCT00338897), or PI-88 which reached phase III trials as an anticancer agent (NCT00268593) and has an antiviral effect against Dengue virus and flavivirus encephalitis [100]. Future *in vitro* studies on the affect that HS moieties have on cell infection by SARS-CoV-2 would be interesting. To conclude, this work has revealed a putative multi-contact binding mechanism of HS to the SARS-CoV-2 spike protein. This highlights alternative ways that HP and HS mimetics could contribute to treatment of COVID-19, other than preventing coagulation and micro-thrombi formation.

## CRediT authorship contribution statement

**Zachariah P. Schuurs:** Conceptualization, Methodology, Software, Formal analysis, Investigation, Data curation, Writing - original draft, Writing - review & editing, Visualization. **Edward Hammond:** Conceptualization, Writing - review & editing. **Stefano Elli:** Formal analysis, Investigation, Writing - review & editing, Investigation. **Timothy R. Rudd:** Formal analysis, Investigation, Writing - review & editing, Investigation. **Courtney J. Mycroft-West:** Methodology, Investigation, Formal analysis, Visualization. **Marcelo A. Lima:** Methodology, Investigation, Formal analysis, Visualization. **Mark A. Skidmore:** Methodology, Investigation, Formal analysis, Visualization. **Richard Karlsson:** Methodology, Investigation, Formal analysis, Visualization. **Yen-Hsi Chen:** Methodology, Investigation, Formal analysis, Visualization. **Ieva Bagdonaite:** Methodology, Investigation, Formal analysis, Visualization. **Zhang Yang:** Methodology, Investigation, Formal analysis, Visualization. **Yassir A. Ahmed:** Methodology, Investigation, Formal analysis, Visualization. **Derek J. Richard:** Writing - review & editing. **Jeremy Turnbul:** Methodology, Validation, Funding acquisition. **Vito Ferro:** Conceptualization, Validation, Writing - review & editing. **Deirdre R. Coombe:** Conceptualization, Validation, Writing - review & editing. **Neha S. Gandhi:** Conceptualization, Methodology, Validation, Formal analysis, Resources, Writing - review & editing, Supervision, Project administration, Funding acquisition.

## Declaration of Competing Interest

The authors declare that they have no known competing financial interests or personal relationships that could have appeared to influence the work reported in this paper.
