# Supplementary material for: Evidence of a putative glycosaminoglycan binding site on the glycosylated SARS-CoV-2 spike protein N-terminal domain
Source: Comput Struct Biotechnol J. 2021 May 4;19:2806–18. doi: 10.1016/j.csbj.2021.05.002 (PMC8093007; doi:10.1016/j.csbj.2021.05.002)
Supplement: Supplementary data 1 [file mmc1.docx]

**Supplementary Data**

**Table S1.** N-linked glycosylations added to asparagine (N) residues on S protein trimer and monomer from Grant et al. [1]. In brackets after each residue number is the number assigned to glycosylation saccharide units.

| 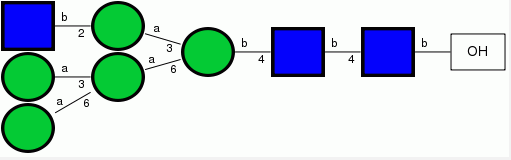 | D-Man*p*α1-6[DMan*p*α1-3]DMan*p*α1-6[DGlc*p*NAcβ1-2DMan*p*α1-3]DMan*p*β1-4DGlc*p*NAcβ1-4DGlc*p*NAcβ1-OH  Residue: 657 (1158-1165) |
| --- | --- |
| 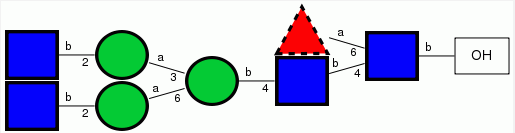 | DGlc*p*NAcβ1-2DMan*p*α1-6[DGlc*p*NAcβ1-2DMan*p*α1-3]DMan*p*β1-4DGlc*p*NAcβ1-4[LFuc*p*α1-6]DGlc*p*NAcβ1-OH  Residues: 149 (1166-1173), 165 (1174-1181), 331 (1182-1189), 343 (1190-1197), 616 (1198-1205), 1134 (1180-1187) |
| 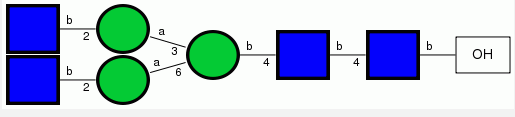 | DGlc*p*NAcβ1-2DMan*p*α1-6[DGlc*p*NAcβ1-2DMan*p*α1-3]DMan*p*β1-4DGlc*p*NAcβ1-4DGlc*p*NAcβ1-OH  Residue: 1098 (1206-1220) |
| 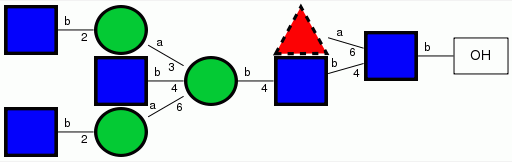 | DGlc*p*NAcβ1-2DMan*p*α1-6[DGlc*p*NAcβ1-4][DGlc*p*NAcβ1-2DMan*p*α1-3]DMan*p*β1-4DGlc*p*NAcβ1-4[LFuc*p*α1-6]DGlc*p*NAcβ1-OH  Residues: 74 (1221-1229), 282 (1230-1238) |
| 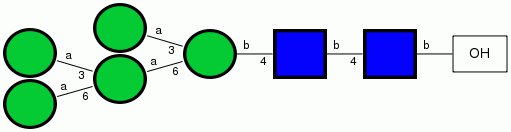 | DMan*p*α1-6[DMan*p*α1-3]DMan*p*α1-6[DMan*p*α1-3]DMan*p*β1-4DGlc*p*NAcβ1-4DGlc*p*NAcβ1-OH  Residues: 61 (1239-1245), 122 (1246-1252), 603 (1253-1259), 709 (1260-1266), 717 (1267-1273), 801 (1274-1280), 1074 (1281-1287) |
| 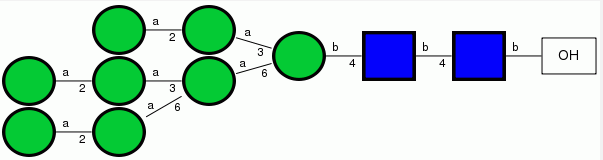 | DMan*p*α1-2DMan*p*α1-6[DMan*p*α1-2DMan*p*α1-3]DMan*p*α1-6[DMan*p*α1-2DMan*p*α1-3]DMan*p*β1-4DGlc*p*NAcβ1-4DGlc*p*NAcβ1-OH  Residue: 234 (1288-1297) |


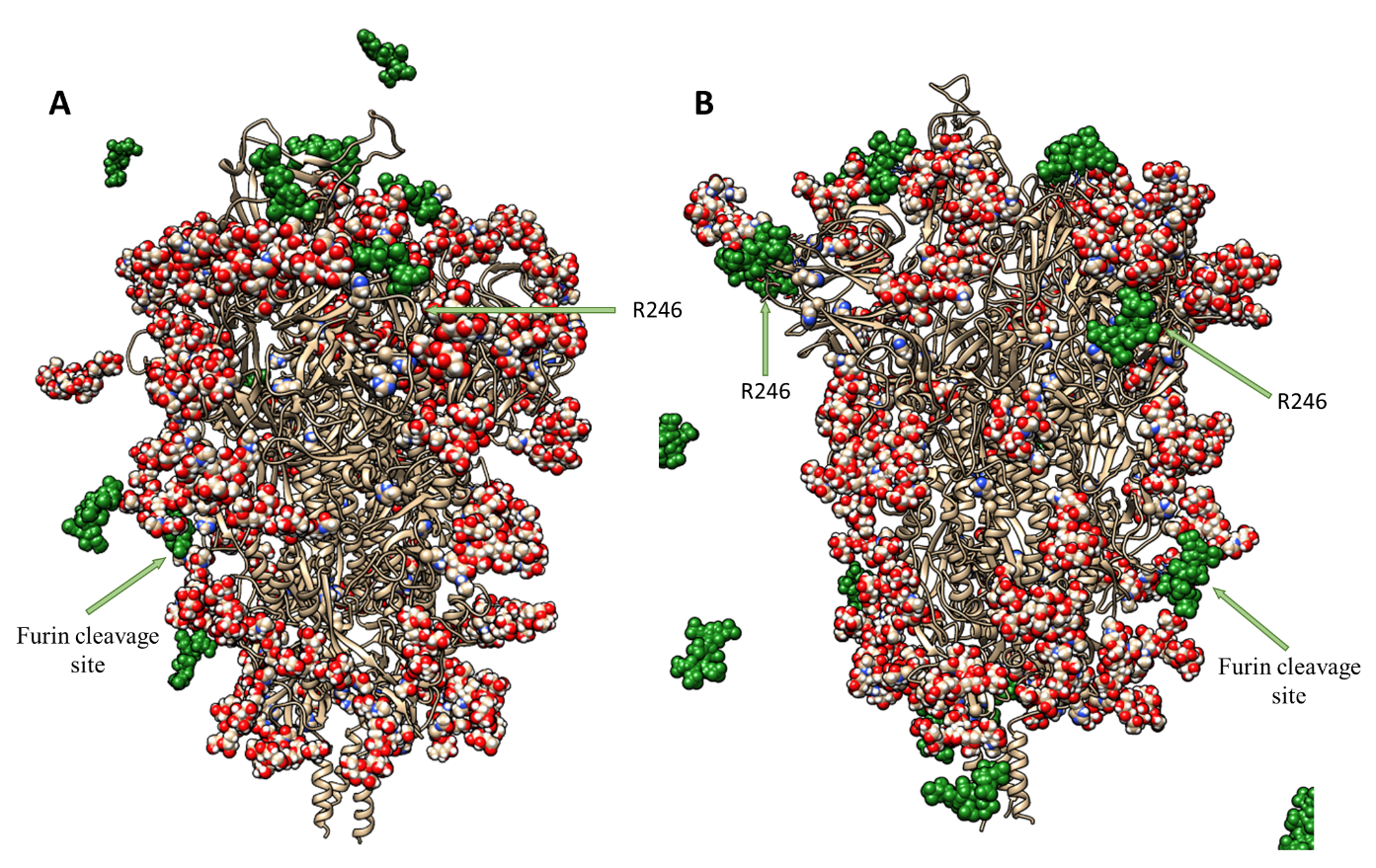


**Figure S1:** Final snapshots of trajectories from further co-solvent MD simulations of the glycosylated trimer system run for 100 ns with ten (A) and twenty (B) HP tetrasaccharide molecules. In both simulations the tetrasaccharides bound to the R246 site and the furin cleavage site.


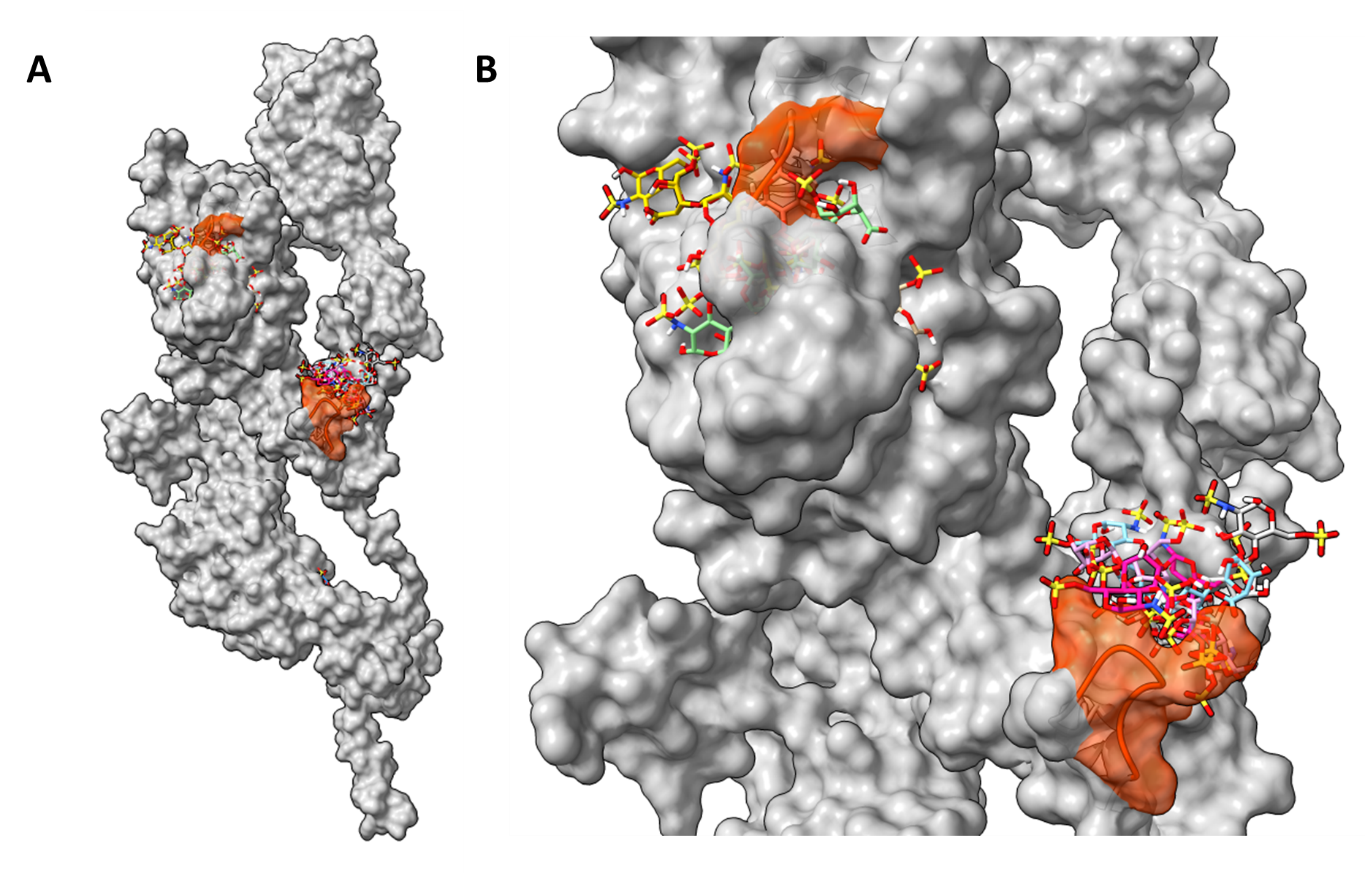


**Figure S2.** Superposed results from ClusPro Server [2] testing binding of heparin tetrasaccharides to the S247R mutated monomer. The sites highlighted in orange are the S247R putative binding region and the PRRAR furin cleavage site. (A) Full length monomer and (B) a close-up of the key binding region.


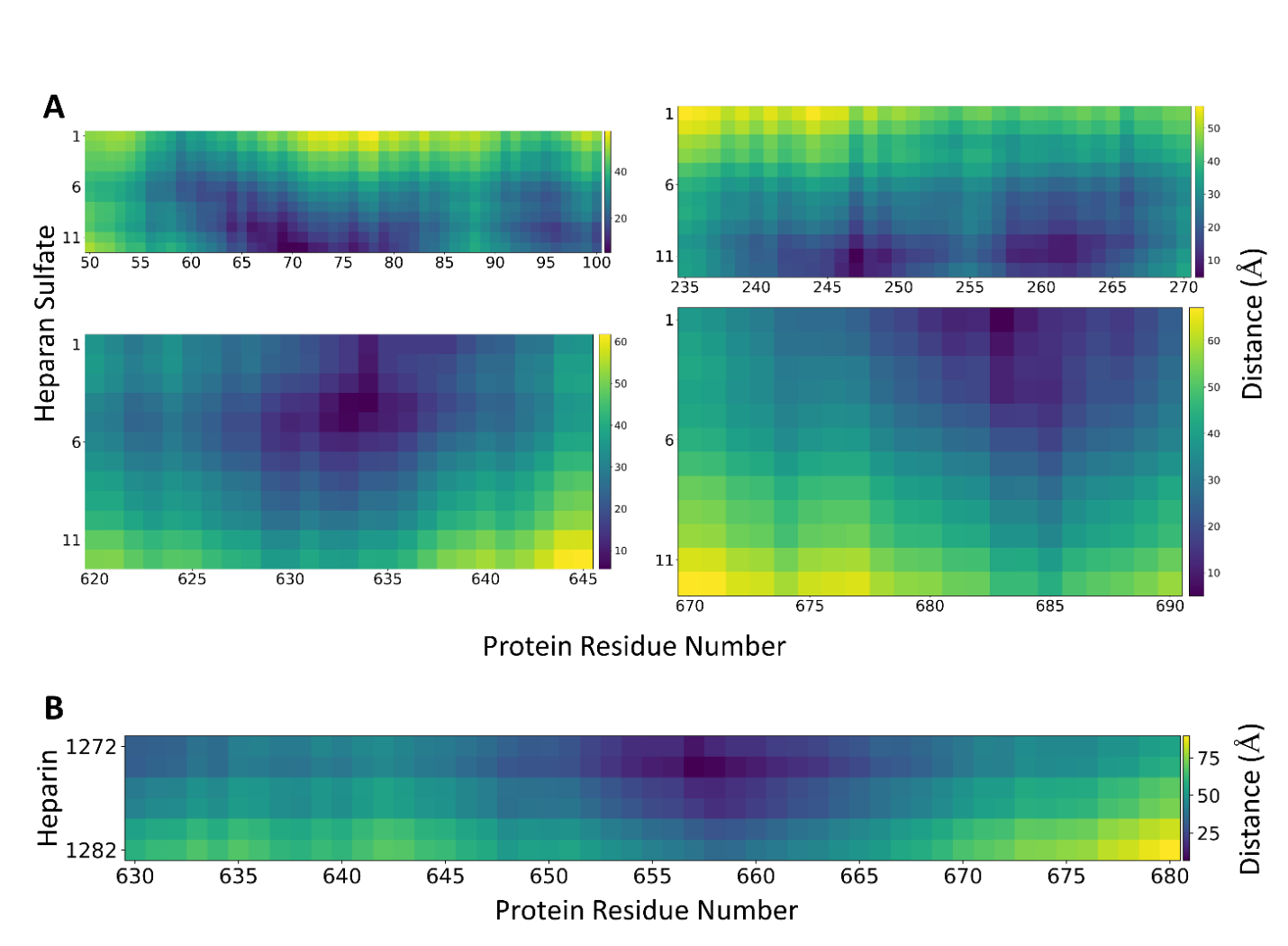


**Figure S3.** Distance to centre of mass of residues (Å) from the SARS-CoV-2 S protein to (A) the HS molecule and (B) the HP molecule. The darker blue indicates two centres of mass being closer.

**Table S2.** Predicted hydrogen bonds between HS and the S protein NTD. Residues and atoms are standard IUPAC-IUB amino acid single letter and atom name nomenclature. The HS subunits in the acceptor column are labelled from 1-12.

| **HS Hydrogen Bonds** | | | | |
| --- | --- | --- | --- | --- |
| **Donor** | | **Acceptor (HS)** | | **Distance (Å)** |
| **Residue** | **Atom** | **Residue** | **Atom** |  |
| H69 | Nδ1 | IdoA2S 12 | OS22 | 1.626 |
| H69 | Nε2 | IdoA2S 10 | O61 | 1.734 |
| S71 | N | IdoA2S 12 | O3 | 2.4 |
| S71 | OG | IdoA2S 12 | O3 | 1.886 |
| R247 | NH1 | GlcNS6S 11 | O61 | 2.41 |
| R247 | NH1 | GlcNS6S 11 | OS63 | 1.949 |
| R247 | NH1 | IdoA2S 12 | O61 | 1.689 |
| A262 | N | GlcNS6S 11 | OS22 | 1.907 |
| R634 | NH1 | IdoA2S 2 | O61 | 1.794 |
| R634 | NH1 | IdoA2S 4 | OS23 | 1.761 |
| R634 | NH2 | IdoA2S 1 | O62 | 1.644 |
| R683 | NH1 | GlcNS6S 1 | OS64 | 1.848 |
| R683 | NH1 | GlcNS6S 2 | O62 | 1.818 |
| R683 | NH2 | GlcNS6S 2 | O61 | 1.727 |

**Table S3.** Predicted hydrogen bonds between HP and the S protein NTD. Residues and atoms are standard IUPAC-IUB amino acid single letter and atom name nomenclature. Note that the glycosylation subunits in the donor residue column also indicate the protein residue (in brackets) that the glycosylation subunit is from. The HP subunits in the acceptor column are labelled from 1-12.

| **HP Hydrogen Bonds** | | | | |
| --- | --- | --- | --- | --- |
| **Donor** | | **Acceptor (HP)** | | **Distance(Å)** |
| **Residue** | **Atom** | **Residue** | **Atom** |  |
| R634 | NH1 | IdoA2S 4 | O61 | 1.54 |
| R634 | NH2 | IdoA2S 4 | O61 | 1.96 |
| R683 | Nε | IdoA2S 8 | OS22 | 1.665 |
| R683 | Nε | IdoA2S 8 | OS24 | 2.865 |
| R683 | NH1 | IdoA2S 6 | O62 | 1.751 |
| R683 | NH2 | IdoA2S 6 | O61 | 1.924 |
| R683 | NH2 | IdoA2S 6 | O62 | 2.446 |
| R685 | NH1 | IdoA2S 8 | OS23 | 1.957 |
| R685 | NH1 | IdoA2S 10 | O62 | 2.067 |
| R685 | NH2 | IdoA2S 10 | O62 | 1.628 |
| Manpβ 1201 (N616) | O4 | IdoA2S 3 | OS64 | 1.889 |
| GlcpNAcβ 1203 (N616) | N | IdoA2S 3 | OS62 | 1.712 |
| Manpα 1201 (N616) | O6 | GlcNS6S 2 | O3 | 1.855 |
| GlcpNAcβ 1205 (N616) | N | GlcNS6S 2 | O3 | 1.848 |
| Manpβ 1241 (N61) | O4 | GlcNS6S 7 | OS64 | 1.76 |
| Manpα 1242 (N61) | O2 | IdoA2S 8 | O5 | 2.732 |
| Manpα 1242 (N61) | O2 | IdoA2S 8 | O62 | 1.949 |
| Manpα 1242 (N61) | O3 | IdoA2S 8 | O62 | 1.996 |
| Manpα 1244 (N61) | O3 | IdoA2S 11 | OS64 | 2.043 |

HS

HP

**Figure S4.** Residue decomposition of the glycosylations added to the S protein that appeared to interact with the ligands. The x-axis numbering are the numbers assigned to glycosylation saccharide units. Refer to **Table S1** for the asparagine residue that the glycosylation structure is attached to. This is a subset of the glycosylations, but it includes those found to interact with the HS and HP ligands. Of particular note are decompositions at 1203, 1205 and 1241-1245.

**Table S4.** Other S protein residues mutated to basic amino acids that could have implications in HS binding. Data from the COVID-19 Viral Genome Analysis Pipeline. Covariations indicate the mutations also occurring in the sequence, Occurrences indicates the number of times these mutations have been picked up in registered viral sequences.

| **Original** | **Residue Number** | **Mutation** | **Covariations** | **Occurrences** |
| --- | --- | --- | --- | --- |
| R | 21 | K | D614G | 3 |
| Y | 145 | H | D614G | 11 |
| Q | 239 | R, H, K | D614G | 15 |
| Q | 675 | R, H, K | D614G | 300 |
| D | 936 | H | D614G | 19 |

**Table S5.** Recent mutations to the SARS-CoV-2 spike glycoprotein that have appeared in Britain and South Africa. Comments have on the mutations that have appeared in each lineage and their potential effect have been made.

| **British B1.1.7 lineage [3]** | | |
| --- | --- | --- |
| **Mutation** | **Region** | **Comment** |
| HV 69-70 deletion | NTD | Histidine directly interacts with HS. Therefore, deletion induces a conformation change and reduces binding[4]. |
| Y144 deletion | NTD | No reported HS/HP interactions. |
| N501Y | RBD | Involved in HP binding, direct interaction with ACE2. |
| A570D | RBD | No reported HS/HP interactions. |
| P681H | PRRARS Cleavage site | R682 involved in direct interaction with HS/HP. Likely that HP chains will interact with P68\1H in a pH dependent manner. |
| T716I | S1 Domain | No reported HS/HP interactions. |
| S982A | HR1 Subdomain |  |
| D1118H | CD Subdomain |  |
| **South African B.1.351 lineage [5]** | | |
| K417N | RBD | Direct interaction with HS/HP[4]. |
| E484K | RBD | Direct ACE2 binding. May affect GAG interactions with S glycoprotein. |
| N501Y | RBD |  |
| L18F | NTD | Not present in spike 3D structures. |
| D80A | NTD | No direct interaction but near 69-70, which are directly involved in HS binding. No drastic affinity change. |
| D215G | NTD | No reported HS/HP interactions. |
| A701V | Central Helix Repeat (S2) |  |
| L242-L244 deletion | NTD | No direct interaction but near the R246 HS binding site. Therefore, deletion will likely induce a conformational change in the loop affecting the binding of HS. |

**References**

(1) Grant O C, Montgomery D, Ito K, Woods R J (2020) Analysis of the SARS-CoV-2 spike protein glycan shield: implications for immune recognition. Sci. Rep.; 10 (14991). http://dx.doi.org/10.1038/s41598-020-71748-7

(2) Mottarella S E, Beglov D, Beglova N, Nugent M A, Kozakov D, et al. (2014) Docking server for the identification of heparin binding sites on proteins. J. Chem. Inf. Model.; 54 (7):2068-78. http://dx.doi.org/10.1021/ci500115j

(3) Rambaut A, Loman N, Pybus O, Barclay W, Barrett J, et al. (2020) *Preliminary genomic characterisation of an emergent SARS-CoV-2 lineage in the UK defined by a novel set of spike mutations*; COVID-19 Genomics Consortium UK: Virological.

(4) Mycroft-West C J, Su D, Pagani I, Rudd T R, Elli S, et al. (2020) Heparin inhibits cellular invasion by SARS-CoV-2: structural dependence of the interaction of the surface protein (spike) S1 receptor binding domain with heparin. Thromb. Haemost.; 120 (12):1700-1715. http://dx.doi.org/10.1055/s-0040-1721319

(5) Tegally H, Wilkinson E, Giovanetti M, Iranzadeh A, Fonseca V, et al. (2020) Emergence and rapid spread of a new severe acute respiratory syndrome-related coronavirus 2 (SARS-CoV-2) lineage with multiple spike mutations in South Africa. medRxiv; Preprint:2020.12.21.20248640. http://dx.doi.org/10.1101/2020.12.21.20248640
